# Fragments of viral surface proteins modulate innate immune responses via formyl peptide receptors

**DOI:** 10.1016/j.isci.2025.113019

**Published:** 2025-06-30

**Authors:** Heiko Heilmann, Lukas Busch, Celine Buchmann, Islam Mohamed, Adrian Theiß, Sabryna Junker, Stefan Lohse, Bernd Bufe

**Affiliations:** 1 Molecular Immunology, Department of Informatics and Microsystems Technology, University of Applied Sciences Kaiserslautern, 66482 Zweibrücken, Germany; 2 Institute of Virology, Saarland University Medical Center, 66421 Homburg, Germany; 3 Institute for Medical Microbiology and Hygiene (IMMH), Saarland University Medical Center, 66421 Homburg, Germany

**Keywords:** Immunity, Immune response, Virology

## Abstract

Formyl peptide receptors (FPRs) are pattern recognition receptors well-known for bacterial pathogen sensing. We here identified activator and inhibitor motifs for FPRs that are present on surface proteins of various viral pathogens. Peptides containing these motifs interact with all FPR family members and modulate various important immune functions in innate immune cells. Viral breakdown products comprising these motifs were found in patients with COVID-19. In the spike protein, many activators are found in highly mutagenic regions, whereas the inhibitor motif is located in a conserved domain that also exists in further unrelated viruses. The physiochemical properties of FPR1 activators correlate with the occurrence of protein aggregation hotspots. Such hotspots are present on various surface proteins of unrelated viruses that can also activate FPRs. This points toward a general contribution of FPRs in modulating antiviral immune responses during many distinct viral infections.

## Highlights

• FPR ligands are present in many different viral surface proteins
• Viral surface protein fragments modulate innate immune responses via FPRs
• Individual FPR subtypes may have a cell type-specific role during viral infection
• Potential FPR interaction sites in viral proteins can be predicted *in silico*

## Graphical abstract

**Figure:**
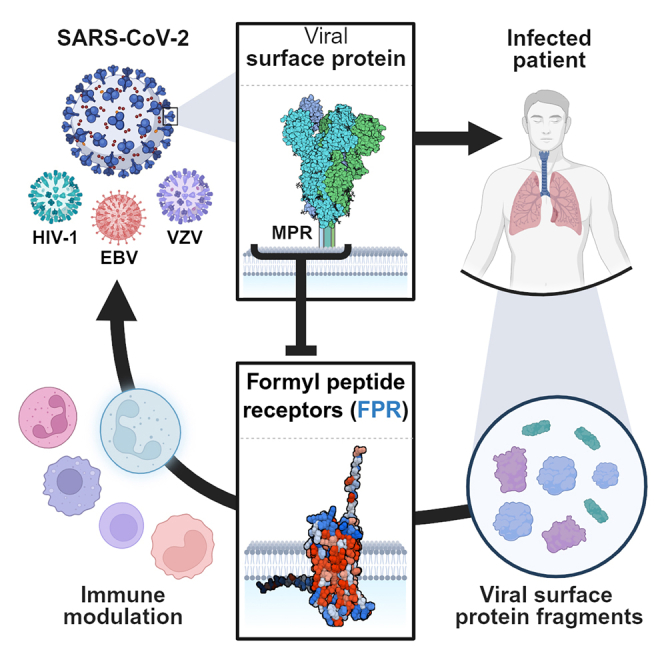

## Introduction

Formyl peptide receptors (FPRs) are a small family of G protein-coupled receptors comprising the three paralog genes *FPR1*, *FPR2,* and *FPR3* in humans.^1^ They are predominantly expressed on the surface of different immune cells, with neutrophils displaying the strongest expression levels.^2^^,^^3^^,^^4^ On immune cells, FPRs serve as well-known sensors for the detection of N-terminally formylated peptides that are either directly produced by invading bacterial pathogens or indirectly released during an infection from damaged mitochondria of necrotic host cells.^5^^,^^6^ Therefore, FPRs were initially seen as pro-inflammatory pattern recognition receptors that play a dual function in innate immunity: the detection of a bacterial pathogen associated molecular pattern (PAMP) and a mitochondrial damage associated molecular pattern (DAMP).^3^^,^^7^^,^^8^^,^^9^ However, further research revealed that the physiological role of FPRs is by far more complex.^5^^,^^10^^,^^11^ While it was initially thought that formylated peptides constitute the sole source for FPR ligands, further research revealed a growing number of additional non-formylated ligands.^1^^,^^12^ These include important host endogenous innate immune modulators such as the antimicrobial peptide LL37, the acute phase protein serum amyloid alpha, and some anti-inflammatory ligands such as annexin A1 (AnxA1) and specialized pro-resolving mediators such as resolvin D2 and lipoxin A4.^2^^,^^12^^,^^13^ In addition, FPRs can interact with aggregation prone neuropathological peptides such as amyloid beta and prion protein.^14^^,^^15^ Furthermore, FPR expression was also found outside the classical innate immune cells in a variety of additional cell types, including microglia,^16^ fibroblasts^17^ and a subset of pain sensitive^18^ and olfactory neurons,^19^ where they contribute to pro- and anti-inflammatory responses,^16^^,^^20^ bacterial infection-related pain perception^18^ and avoidance of infected conspecifics.^21^ More recently, some evidence for a role of FPRs in viral infections has been raised.^1^^,^^2^^,^^7^^,^^9^^,^^11^ The best examined case is the influenza A virus (IAV) infection,^22^^,^^23^^,^^24^^,^^25^ where AnxA1-and mFpr2-deficient mice both showed a significantly increased survival rate,^26^ which clearly demonstrates that an AnxA1/FPR2 interaction promotes IAV infections. Another very recent example where AnxA1/FPR2 interactions contribute to a viral pathology is the dengue virus (DENV).^27^ However, in this case, the AnxA1/FPR2 interaction plays a protective role because the absence of either FPR2 or AnxA1 led to disease deterioration and exacerbated inflammation, whereas the application of the FPR2 agonist Ac_2-26_ which corresponds to the N-terminal AnxA1 domain, alleviated disease severity.^27^ Next, FPRs may interact with other viral surface proteins. In heterologous expression systems, FPR1 and FPR2 bound peptides derived from the human HIV-1 glycoproteins gp120 and gp41.^28^^,^^29^^,^^30^^,^^31^^,^^32^^,^^33^ Moreover, FPR2 can also act as a co-receptor for several primary isolated HIV-1 strains in cell culture experiments.^34^ The FPR *in vitro* ligand T20/DP178 that originates from the highly conserved, c-terminally tryptophan rich region of gp41 was even used to develop a fusion inhibitor that could effectively block HIV infections *in vitro* and as an approved drug *in vivo.*^35^^,^^36^ Interestingly, the C-terminal part of T20/DP178 that is known as the membrane proximal region (MPR) has analogous sequences in a number of different viruses such as ebola virus, common cold coronaviruses and severe acute respiratory syndrome coronavirus (SARS-CoV) that interact with FPR1.^37^^,^^38^ Peptides from MPR region of different coronavirus strains were shown to suppress FPR1 downstream signaling and cell surface expression^37^ in an heterologous expression system and in another context, recombinant spike protein fragments have recently been shown to induce signaling in cardiovascular cells.^39^^,^^40^ Taken together, these findings raise the possibility that viral break down products may modulate immune cell signaling via FPRs. This might help to explain some of the complex pathophysiological responses observed during COVID-19 infections and post-COVID syndrome. However, systematic studies examining the link between FPRs, the SARS-CoV-2 break down products, and the responses of innate immune cells are still lacking.

We here first assessed publicly available single cell RNA sequencing metadata and found an upregulation of all FPR subtypes in many disease-relevant cell types of lung tissue from patients with COVID-19. Next, we found that MPR peptides of SARS-CoV-2 and several other common cold coronaviruses displayed an evolutionarily conserved inhibition of FPR1-mediated calcium signaling, but were also able to activate FPR2 and FPR3. Experiments with primary isolated human neutrophils that co-express FPR1 and FPR2 demonstrated that the FPR1-associated inhibition of calcium signaling by MPR peptides prevails over FPR2 activation. We also observed that some peptide fragments can modulate migration, MMP-9 release, NETosis, and ERK1/2 signaling. By using a peptide pool that comprised 80 peptides from hypervariable regions of the spike protein ectodomain, we found many additional peptides from at least seven regions within the spike protein that triggered calcium flux of all three human FPRs and primary isolated human neutrophils. Of note, peptide fragments and corresponding IgG epitopes of several peptides were found in patients with COVID-19. Furthermore, an analysis of the physiochemical properties of these viral FPR activators revealed that FPR1 favors hydrophobic peptides that contain aggregation hotspots which indicate protein-protein interaction sites. As similar hotspots are present in many viral surface proteins, we tested and confirmed that envelope protein fragments from multiple unrelated viruses could also activate FPRs. This raises new insights into the molecular recognition mechanism that forms the basis for the fascinatingly broad but highly specific detection capabilities of FPRs.

## Results

### Membrane proximal region peptides show an evolutionary conserved interaction with all human formyl peptide receptors

**Figure 1:**
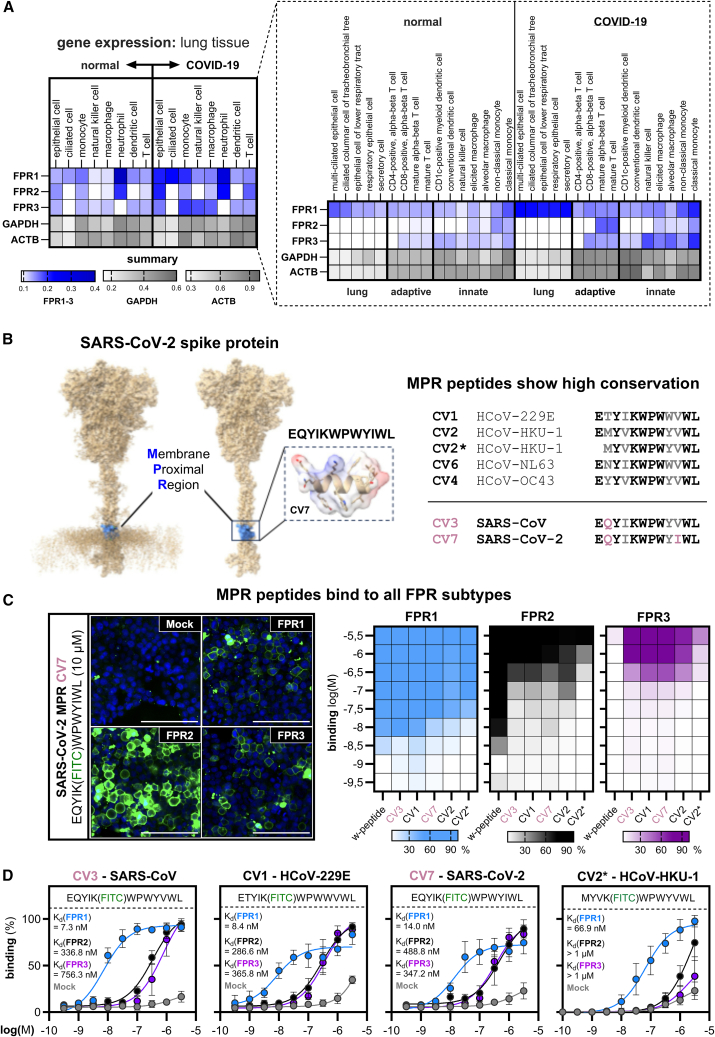
Evolutionary conserved interaction of peptides from the membrane proximal region of different *coronaviridae* species with FPR1, FPR2, and FPR3 (A) Single-cell RNA sequencing metadata for FPR expression in lung tissue samples from patients with COVID-19 for selected cell types in comparison to normal, healthy control. Expression data for target genes *FPR1-3* and housekeeping genes *GAPDH* and *ACTB* were gathered from the CELLxGENE repository and comprise 24 individual, published datasets.^42^ (B) Left: SARS-CoV-2 spike protein model^45^ visualizing membrane proximal region (MPR) peptides in the SARS-CoV-2 spike protein. Right: conservation between MPR peptides of different coronavirus strains.^37^ Sequence is shown in one letter amino acid code. Conserved residues are shown in black, variable positions in gray, deviating residues of SARS-CoV and SARS-CoV-2 compared to the other common cold coronaviruses are shown in lilac. (C) Left: representative confocal images of the binding of FITC-labeled SARS-CoV-2 MPR peptide CV7 on HEK293T cells transfected with either Mock, FPR1, FPR2, or FPR3. Scale bar represents 100 μm. Right: comparison of the binding affinities of the different fluorescently labeled peptides W-peptide, CV3 (SARS-CoV), CV7 (SARS-CoV-2), CV1 (HCoV-229E), CV2 (HCoV-HKU-1), and CV2∗ (HCoV-HKU-1) on transfected HEK293T cells. (D) Binding kinetics of the fluorescently labeled MPR peptides as described in (C). Data are represented as mean +/− standard deviation (SD) from at least three independent experiments (*n* ≥ 3) with three visual fields (*N* = 3) that were normalized to the largest value of the respective dataset. See also Figures S1 and S2.

FPR1 has recently been proposed as a potential target for assessing COVID-19 disease-mediated inflammation and disease severity in patients.^41^ To examine if COVID-19 infections induce changes in the expression of different FPR genes*,* we first performed an analysis of publicly available single-cell RNA sequencing data from 24 independent COVID-19 studies^42^ (Figure 1A). We observed a general upregulation in mRNA expression levels for all *FPR* subtypes in lung tissue of infected patients in many disease-relevant cell types such as monocytes, epithelial cells, ciliated cells, and T-cells. *FPR1* displays a prominent upregulation in epithelial cells. *FPR2* is upregulated in T-cells that regulate the adaptive immune response, while *FPR3* expression is increased in innate immune cells such as macrophages and monocytes. Of note, *FPR1* expression in lung epithelial cells and further lung cell types has been reported to triggers wound-healing, leukocyte infiltration, and smoke-induced inflammation upon stimulation.^43^^,^^44^ *FPR2,* on the other hand, has not been reported to be expressed in any of the listed cell types; however, it can adapt an either beneficial or deleterious role in lung cell types such as nasal and bronchial epithelial cells.^44^ Interestingly, an investigation of *FPR* gene expression in peripheral blood mononuclear cells (PBMC) of SARS-CoV-infected patients also showed similar upregulation of *FPR1* and *FPR2* (Figure S1). In summary, these data support the previous findings and raise the possibility for a cell type-specific role of the individual FPR subtypes during coronavirus infections.

Several peptides from the membrane proximal region (MPR) of different common cold-inducing *coronaviridae* species such as HCoV-229E, HKU1, NL63 and OC43 (Figure 1B) were previously shown to interact with FPR1.^37^ However, their ability to interact with other FPRs is not known. We therefore aimed at investigating the interaction of FPR2 and FPR3 with peptides from the membrane proximal region (MPR). As the MPR domain is evolutionary conserved, it is also present in the spike protein of SARS-CoV and SARS-CoV-2. A sequence comparison of the common cold-inducing coronavirus MPR peptides with the corresponding SARS-CoV and SARS-CoV-2 domains revealed that both SARS-CoV variants contain a glutamine residue at the second position that is not present in all other strains (Figure 1B). Thus, we wondered how this alteration would affect interactions with FPR1, FPR2, and FPR3. By utilizing transiently transfected HEK293T cells in high-throughput confocal microscopy, we found that the FITC-labeled MPR peptide CV7 from SARS-CoV-2 was capable of binding to FPR1, FPR2, and FPR3 with K_d_ values of 14 nM, 488 nM, and 347 nM, respectively (Figures 1C and 1D). Similar results were obtained for MPR peptides derived from SARS-CoV (CV3), HCoV-229E (CV1), and HCoV-HKU-1 (CV2, Figure S2). Despite some variations in their amino acid sequence, these MPR peptides displayed similar binding sensitivity. However, the binding affinity for FPR1, FPR2, and FPR3 toward the N-terminally truncated MPR peptide CV2∗ from HCoV-HKU-1 was approximately 10-fold lower, indicating that variations in the N-terminus could critically affect interactions with FPRs. Taken together, our data demonstrate a conserved interaction of MPR peptides of both SARS-CoV variants and other *coronaviridae* species with all FPR subtypes, which argues for a fairly stable interaction of FPRs and MPR peptides throughout coronavirus evolution. While FPR1 is the most sensitive interaction partner with binding affinities in the low nanomolar range, FPR2 and FPR3 can also interact with MPR peptides at approximately 50- to 100-fold higher concentrations.

### Peptides from the membrane proximal region of coronaviruses inhibit calcium signaling and neutrophil immune cell responses

**Figure 2:**
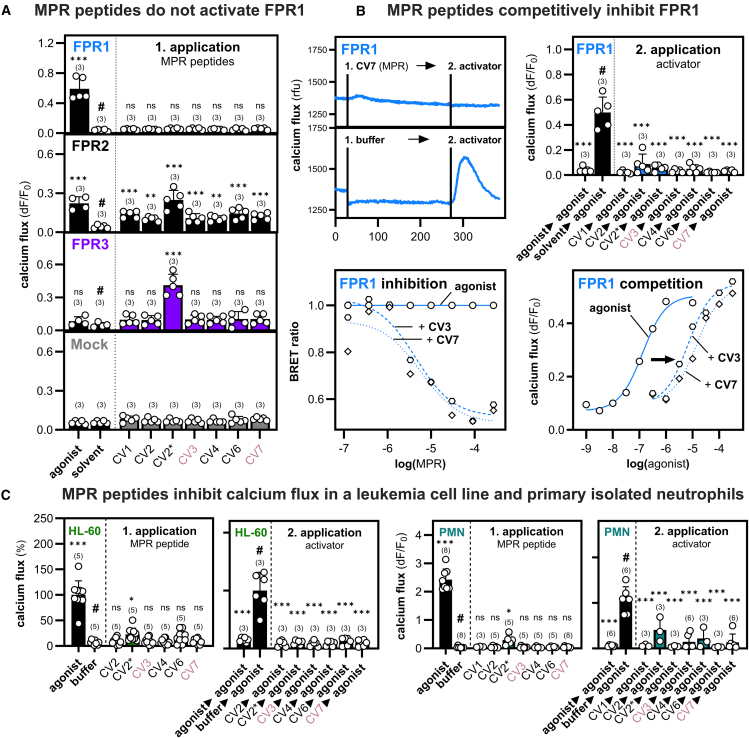
MPR peptides competitively inhibit FPR-depended calcium flux in human neutrophils (A) Mean calcium responses (*n* = 3) of HEK293T cells transfected with FPR1, FPR2, FPR3, or Mock plasmid toward MPR peptides from different coronaviruses (30 μM). Agonist denotes the FPR1/2 activator W-peptide (10 μM), solvent denotes buffer with added DMSO as used in peptide dilution. (B) Upper left: representative calcium traces for two consecutive ligand applications that demonstrate the inhibitory effect of SARS-CoV-2 (10 μM) on the response of FPR1 transfected HEK293T cells toward the classical FPR activator W-peptide (10 nM). Upper right: mean calcium signals for W-peptide (10 nM) after a prior application of 10 μM of the indicated MPR peptides (*n* = 3). Lower left: displacement curve of f-MVPIK (TAMRA)I; (50 nM) upon co-application with CV3 and CV7 in a bioluminescence resonance energy transfer (BRET) assay (*n* = 3). Lower right: competitive inhibition of the calcium release of FPR1 transfected HEK293T cells upon the co-application of varying concentrations of W-peptide in the presence of 10 μM CV3 or CV7 (*n* = 4). (C) Mean calcium responses of neutrophil-like differentiated HL-60 cells (dHL-60) or human polymorphonuclear granulocytes (PMN; neutrophils) after an initial and a consecutive ligand application. This demonstrates the inhibitory effect of CV peptides (10 μM) on the activation of agonist W-peptide that was used at 100 nM for dHL60 (n = 3–5) and at 10 nM for neutrophils (n = 3–8). Data are represented as mean +/− SD. Statistics were performed using Dunnett’s post-hoc one-way ANOVA to a reference value that is indicated by #. Significances are ∗*p* ≤ 0.05; ∗∗*p* ≤ 0.01; ∗∗∗*p* ≤ 0.001; ns, no significance.

To understand the effects of MPR ligands on the signal transduction of the individual FPRs, we initially screened different MPR peptides for their activity on HEK293T cells transfected with human FPR1, FPR2, or FPR3 using high-throughput calcium imaging (Figure 2A). Consistent with a previous report^37^ claiming that these peptides do not trigger G-protein activity in FPR1 transfected cells, the application of MPR peptides did not induce calcium flux in FPR1-expressing HEK293T cells for any of the tested peptides. Surprisingly, we observed a significant elevation of calcium levels in FPR2 transfected cells for all tested peptides. In contrast, FPR3-transfected cells only responded to the CV2∗ peptide that lacks an N-terminal glutamate, whereas the full length CV2 peptide was not capable of eliciting any signal. This indicates that slight alterations in the ligand structure can already affect the pattern of activated FPRs. We next tested whether MPR peptides may act as an inhibitor (Figure 2B). Indeed, MPR peptide application, followed by a subsequent application of a potent FPR1 activator, completely abolished the calcium flux that is usually seen with this activator. Binding experiments using bioluminescence resonance energy transfer (BRET) and calcium signaling experiments in which we simultaneously co-applied the MPR peptides with an agonist revealed that MPR peptides act as competitive inhibitors. FPR1 inhibition starts approximately at a concentration of 1 μM of the MPR peptides. The IC_50_ values for CV3 and CV7 from both SARS-CoV variants in the BRET assay were 3.8 μM and 5.1 μM, respectively. In calcium imaging experiments, the co-application of increasing W-peptide concentrations in the presence of CV3 derived from SARS-CoV (10 μM) or CV7 derived from SARS-CoV-2 (10 μM) led to a 52- and 109-fold rightward shift of the EC_50_ values, respectively. In calcium imaging experiments, the FPR1 EC_50_ value of agonist W-peptide (131.3 nM) shifted by approximately 100-fold to 14.2 μM in the presence of CV7. Similar EC_50_-shifts were obtained in the presence of CV2∗ (EC_50_ = 1.9 μM), CV1 (EC_50_ = 2.7 μM), CV3 (EC_50_ = 6.8 μM), CV4 (EC_50_ = 7.1 μM).

In innate immune cells, FPR1 and FPR2 are frequently co-expressed.^4^ We therefore wanted to know how the opposing effects of MPR peptides on FPR1 and FPR2 would affect innate immune cell behavior. We examined this question by using differentiated HL-60 cells (dHL-60) that represent a well-established *in vitro* neutrophil-like cell model as well as primary isolated human polymorphonuclear leukocytes (PMNs), with neutrophils representing the predominant fraction (Figure 2C). The full-length MPR peptides did not elicit any significant calcium signal in dHL-60 cells, and a subsequent agonist application did not trigger any calcium flux. The only exception was a positive calcium flux induced by the N-terminally truncated CV2∗ peptide, which showed the strongest FPR2 and FPR3 activation. Similar effects were observed in primary isolated human neutrophils, which are known for their high expression levels of FPR1 and FPR2.^3^ Taken together, these results fully confirm all data observed for the individual receptors in our heterologous expression systems and demonstrate that the competitive inhibition effect of MPR peptides on FPR1 in calcium signaling is likewise seen in primary neutrophils.

### Identification of key FPR1 interaction sites in MPR peptides

**Figure 3:**
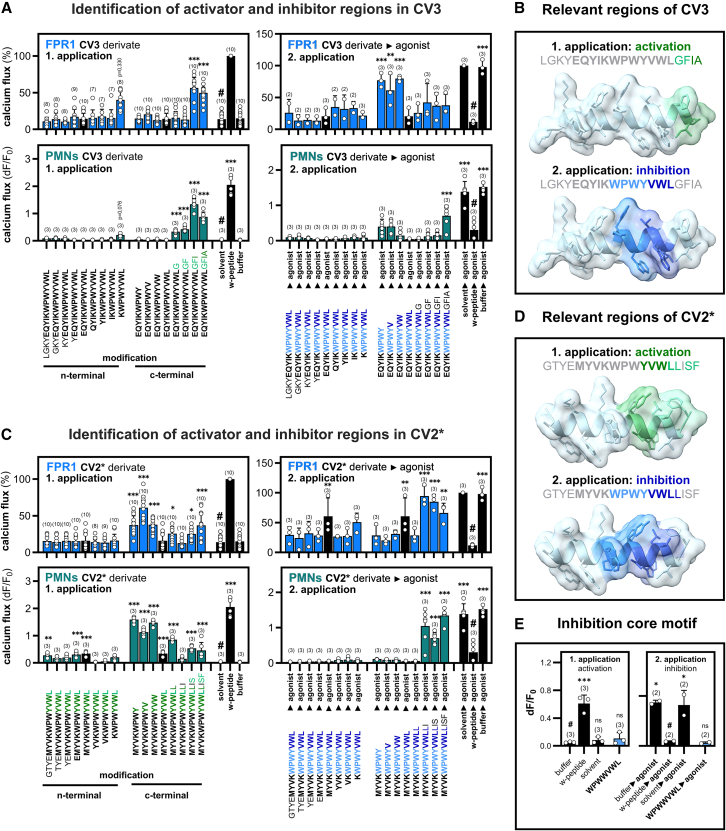
Identification of key regions in MPR peptides for FPR1-mediated neutrophil inhibition and activation (A) Upper left: activation pattern of the systematically extended or truncated SARS-CoV MPR peptide CV3 (10 μM) on FPR1 transfected HEK293T cells (n = 7–10). Lower left: activation pattern of the same peptides on human polymorphonuclear granulocytes (PMN; neutrophils) (*n* = 3; *N* = 2). Upper right: inhibition pattern of a subsequent application of agonist W-peptide (50 nM) (*n* = 3). Lower right: inhibition pattern of neutrophils toward the subsequent application of agonist W-peptide (10 nM) (*n* = 3; *N* = 2). (B) Visualization of inhibitor and activator regions of CV3. (C) Upper left: activation pattern of the systematically extended or truncated MPR peptide CV2∗ (10 μM) on FPR1 transfected HEK293T cells (n = 7–10). Lower left: activation pattern of the same peptides on neutrophils (*n* = 3; *N* = 2). Upper right: inhibition pattern toward a subsequent application of agonist W-peptide (50 nM) (*n* = 3). Lower right: inhibition pattern of neutrophils toward the subsequent application of agonist W-peptide (10 nM) (*n* = 3; *N* = 2). (D) Visualization of inhibitor and activator regions of CV2∗. (E) Left: calcium response of HEK293T cells transfected with FPR1 toward the inhibition core motif of CV6 (*n* = 3). Right: calcium response toward the subsequent application of agonist W-peptide (100 nM) (*n* = 2). Data are represented as mean +/− SD. The application of FPR agonist W-peptide (10 μM) was used as a positive control for activation, and the solvent negative control represents buffer with added DMSO as used in peptide dilution. Statistics were performed using Dunnett’s post-hoc one-way ANOVA to a reference value that is indicated by #. Significances are ∗*p* ≤ 0.05; ∗∗*p* ≤ 0.01; ∗∗∗*p* ≤ 0.001; ns, no significance. See also Figure S3.

Coronavirus infections trigger the release of different types of proteases that can lead to the degradation of the spike protein.^46^^,^^47^^,^^48^ Thus, multiple different types of peptide fragments containing the MPR region can occur. Therefore, we next systematically investigated the impact of N- and C-terminally elongated or truncated peptide derivatives from the SARS-CoV MPR peptide CV3 and the HCoV-HKU-1 MPR peptide CV2∗ on FPR signaling. For that, we first tested whether the peptide derivatives can lead to an activation of FPR1-transfected HEK293T cells (Figure 3A). Neither N-terminal elongation nor truncation by up to four residues nor C-terminal truncation of up to three residues of CV3 triggered any calcium responses, whereas we surprisingly observed that a C-terminal extension of more than two residues would lead to an activation of FPR1. We then tested the inhibitory properties of CV3 by a consecutive application of the FPR agonist W-peptide. N-terminal extensions or truncations by up to four residues and C-terminal extensions of up to four residues did not affect the inhibitory properties. In contrast, a C-terminal truncation by more than one residue completely abolished the inhibitory effect. This shows that the C-terminus is critical for FPR interactions. Next, we investigated how the peptides modulate the responses of primary human neutrophils. The activation response pattern of neutrophils to the CV3 derivatives was similar to the one observed for FPR1 transfected HEK293T cells. In line with our previous results obtained for HEK293T cells, N-terminal elongation or truncation did not induce any activation. However, we observed that neutrophils were more sensitive to C-terminal elongations. The inhibitory effect of CV3 derivatives on neutrophil calcium signaling was even more pronounced than on FPR1 transfected HEK293T cells. Taken together, these data prove the existence of two functional domains within the MPR domain of SARS-CoV: a stronger inhibitory WPWYVWL sequence in the middle of the CV3 peptide and a weaker modulatory activator sequence GFIA at the very C-terminus (Figure 3B).

Interestingly, analog tests with the peptide CV2∗ revealed that the activator motif can vary between different coronavirus strains. The inhibitory site WPWYVWLL is nearly identical to CV3 with the exception that the CV2∗ motive comprises an additional C-terminal leucine residue. The activator sequence differs significantly as it comprises a YVW core sequence and is modulated by the LLISF sequence at the C-terminus (Figures 3C and 3D). These data indicate that the core motif for binding and inhibition is included in the WPWYVWL region. The corresponding sequence domain of the HCoV-NL63 MPR peptide CV6, which contains the slightly deviating sequence WPWWVWL, provided evidence that small variations in this sequence can be tolerated (Figure 3E). In addition, the results with neutrophils demonstrated that a C-terminal extension can turn the inhibitor into an activator (Figure 3C). The CV2∗ peptide that lacks an N-terminal glutamic acid triggers ERK signaling via FPR1 and neutrophil migration (Figure S3) and FPR3 activation (Figure 2A). However, the full-length CV2 peptide (Figure 1B) and all other tested MPR peptides that contain this glutamic acid did not induce these effects (Figure S3). Thus, the length of MPR peptides and precise sequence can significantly influence the resulting signal transduction and innate immune responses.

### Multiple spike protein ectodomain peptides are potent formyl peptide receptor activators

**Figure 4:**
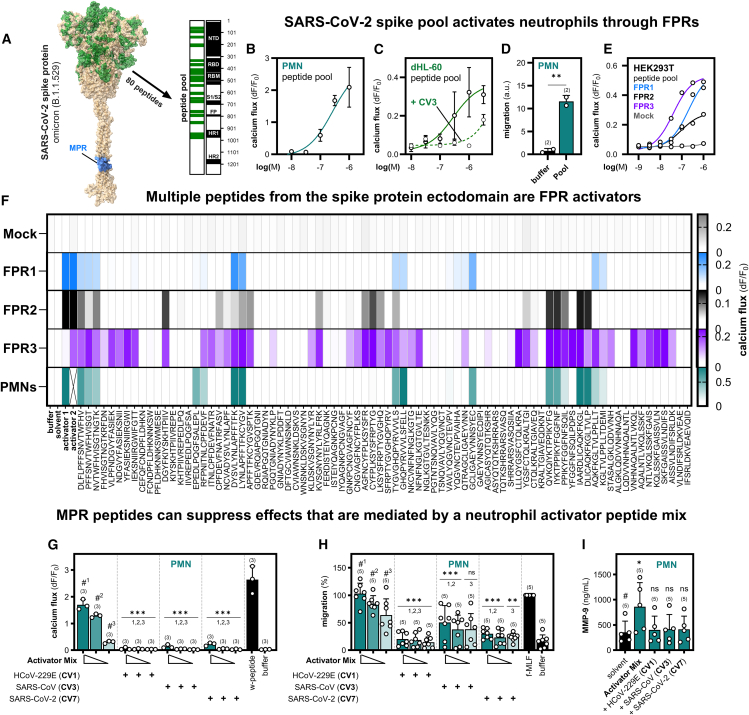
The SARS-CoV-2 spike protein contains many FPR activators that can be inhibited by MPR peptides (A) Visualization of the peptide distribution in the ectodomain of the referenced Omicron mutation spike peptide pool in a SARS-CoV-2 spike protein model^45^ (analog to P0DTC2). (B) Dose-dependent calcium response of human polymorphonuclear granulocytes (PMN; neutrophils) to the Omicron mutation spike peptide pool (*n* = 3). (C) Dose-dependent calcium response of neutrophil-like differentiated HL-60 cells (dHL-60) to the peptide pool with (n = 3–4) and without (*n* = 2) the addition of SARS-CoV MPR peptide CV3 (10 μM). (D) Neutrophil migration in response to the peptide pool (1 μM) (*n* = 2). (E) Calcium response of HEK293T cells transfected with either FPR1, FPR2, FPR3 or Mock toward the peptide pool (*n* = 3). (F) Heatmap of the calcium responses of transfected HEK293T cells (Mock, FPR1, FPR2, FPR3) (*n* = 3) and neutrophils (*n* = 6) toward a library of the 80 individual peptides (10 μM) originating from the Omicron mutation spike peptide pool. Negative controls represent the appropriate assay buffer (PMNs, Ringer solution; HEK293T, C1 assay buffer) and solvent (DMSO in equal amounts to ligand dilution). Activator 1 (W-peptide, 10 μM) served as a positive control for FPR1/FPR2 transfected HEK293T cells and neutrophils. Activator 2 denotes the FPR3 activator WKYMVm-CHO (10 μM; not tested on neutrophils).^18^^,^^54^ (G) Calcium response of neutrophils to a mixture of 10 selected FPR activators from the Omicron mutation spike peptide pool that were tested in three different concentrations (3 μM, dark teal, 1 μM teal, 300 nM light teal) (*n* = 3). MPR peptides HCoV-229E (CV1), SARS-CoV (CV3) and SARS-CoV-2 (CV7) were co-applied in a concentration of 10 μM. Negative control: buffer; positive control: W-peptide (10 μM). (H) Corresponding experiments for neutrophil migration (*n* = 5). Negative control: buffer; positive control: f-MLF (10 nM). (I) MMP-9 protease release for the activator mix (3 μM) in neutrophils (*n* = 5). Negative control: buffer; positive control: W-peptide (10 μM). Data are represented as mean ± SD. Statistics were performed using unpaired t-test (C), Dunnett’s post-hoc one-way ANOVA (G + H), or Mann-Whitney test (I) to a reference value that is indicated by # (numbered in case of multiple references). Significances are ∗*p* ≤ 0.05; ∗∗*p* ≤ 0.01; ∗∗∗*p* ≤ 0.001; ns, no significance. See also Figures S4, S5, and S6.

Interactions between FPRs and peptide derivatives from distinct envelope protein domains in other viruses have already been described.^28^^,^^29^^,^^31^^,^^33^ Therefore, we wondered whether there might be additional FPR interaction partners in regions of the SARS-CoV spike protein ectodomain besides the MPR domain. To address this question, we tested a peptide library that comprises 80 peptides derived from several infection- and evolution-relevant sites in the SARS-CoV-2 Omicron B.1.1.529 variant spike protein^49^^,^^50^ (Figure 4A). To investigate if these peptides can trigger innate immune responses, we first challenged primary isolated human neutrophils and neutrophil-like dHL-60 cells with the pooled peptides. Here, we observed a concentration-dependent calcium response (Figures 4B and 4C) and a robust migration (Figure 4D). Similar effects could be observed in response to a second peptide pool comprising peptides that exclusively cover the receptor binding domain (RBD) of SARS-CoV-2 Omicron B.1.1.529 (Figure S4). We then used the dHL-60 cell model to test if MPR peptides can interfere with these calcium responses. In line with our expectations, the co-application of the MPR peptide CV3 and the pooled peptides competitively inhibited the calcium signals (Figure 4C). Next, we used transfected HEK293T cells to test which receptor subtypes would be activated by the peptide pool. Surprisingly, we observed calcium signaling by all three receptors in low micromolar concentrations (Figure 4E). Hence, we wanted to know which peptides caused the activation of the respective receptor subtypes. To this end, we created a peptide library that covers all 80 peptides of the SARS-CoV-2 omicron B.1.1.529 spike protein peptide pool as individual 15mer peptides (partially overlapping by four residues). Using high-throughput calcium imaging, we screened each of the individual peptides on FPR-transfected HEK293T cells and human neutrophils (Figures 4F and S5A). We identified 10 activators for FPR1, 9 for FPR2, and 30 for FPR3. Intriguingly, 19 of the FPR3 activators were specific for this receptor subtype and did not activate FPR1 or FPR2. Further tests with the FPR3 activators revealed that two peptides even displayed nanomolar EC_50_ values (Figure S6). This observation was particularly surprising, because the total amount of known agonists for the human FPR3 receptor is extremely low. To date, in addition to a few FPR2/FPR3 activators such as Humanin^51^^,^^52^ only one preferential FPR3 activator F2L^53^ is known. We therefore find the enormous quantity of specific FPR3 activators very remarkable. Next, we analyzed the effect of the individual peptides on primary isolated human neutrophils. Here, we observed a statistically significant calcium mobilization for 13 peptides from 7 different spike protein regions (Figures 4F and S5A). To see if these signals are FPR-dependent, we challenged the 12 strongest neutrophil activators with the competitive FPR1/2-inhibitor t-Boc2 (Figure S5B). As expected, we observed marked inhibition of the neutrophil responses in all 12 cases, which demonstrated that the signaling was dependent on FPRs. However, we also noticed that the activation pattern of FPR1 correlated well with neutrophil calcium signals, while there was no clear correlation of FPR2 and FPR3 activation with the neutrophil response pattern. Moreover, we observed that a number of FPR2 and/or FPR3 selective activators did not induce any calcium signal in human neutrophils, suggesting that the responses are most likely FPR1-dependent. Further experiments with 10 of the peptides that activated PMNs revealed that two of them could trigger NETosis (Figure S5C), which argues that these peptides can potentially modulate a variety of different immune functions. Lastly, we tested the effect of MPR peptides on the ectodomain activator-induced responses in neutrophils. For these experiments, we administered a mixture of the ten strongest activators at three different concentrations (3 μM, 1 μM, and 0.3 μM) in the presence of one of the three MPR peptides CV1, CV3, or CV7 (Figure 4G). As expected, the co-application of the mix with any of the MPR peptides led to a calcium flux suppression. Further tests revealed that the activator peptide mix can also trigger chemotactic migration (Figure 4H). Again, the co-application of the peptide mixture with CV1 or CV7 led to a near complete inhibition of the migratory response, whereas CV3 displayed only partial inhibition. Likewise, the ectodomain activator triggered the release of matrix metalloprotease 9 (MMP-9), which again could be attenuated by any of the tested MPR peptides (Figure 4I). Taken together, these activator peptides can trigger a number of important innate immune responses, which are, however, modulated by MPR peptides.

### Envelope protein fragments from various viruses can activate all human formyl peptide receptors

**Figure 5:**
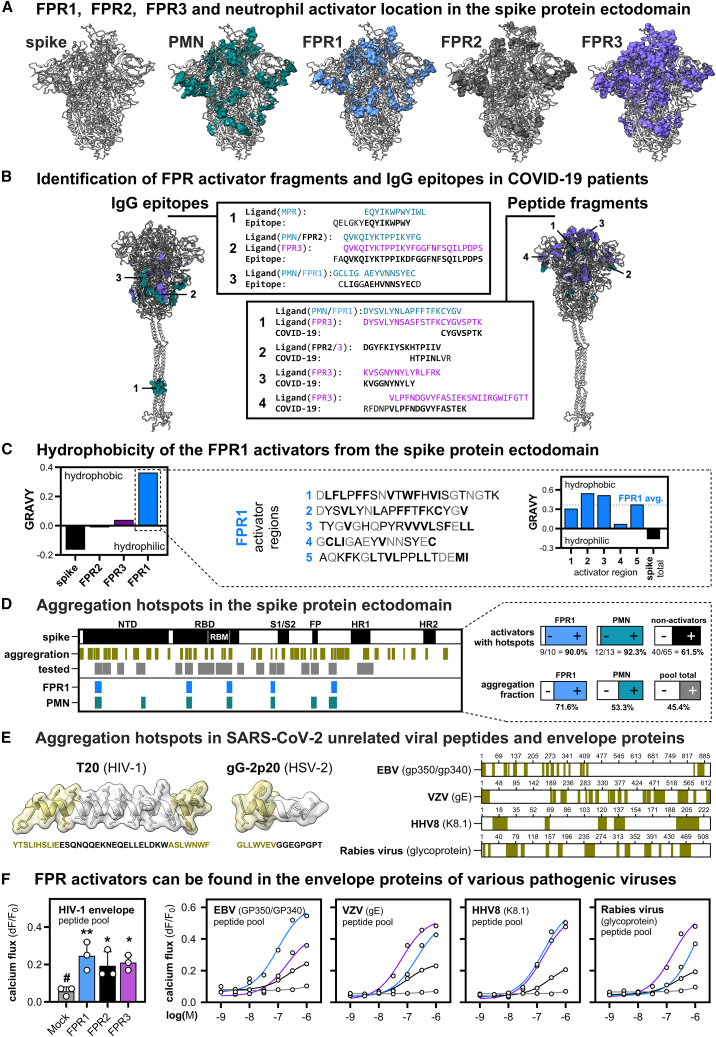
Aggregation hotspots in various viral envelope proteins correlate with the activity of FPR1 (A) Visualization of all calcium activators found for FPR1, FPR2, FPR3, and PMNs from the peptide screening in the SARS-CoV-2 Omicron Spike B.1.1.529 (PDB: 7QO7). (B) Identified peptides and verified IgG epitopes in patients with SARS-CoV-2 shown with respect to their location in the spike protein^45^ (analog to UniProt: P0DTC2). Marked are the peptides and epitopes with the best sequence match, all sequences are listed in Table S2. (C) Left: analysis of the amino acid sequence hydrophobicity from the FPR1, FPR2, or FPR3 activator regions in comparison to the whole spike protein. Right: hydrophobicity of the individual FPR1 activator regions. Color code of amino acids represents hydrophobic residues (black, bold) or non-hydrophobic residues (gray). (D) Left: Alignment of protein aggregation hotspots, the tested fraction that was covered by the peptide screening as well as the identified FPR1 and neutrophil activators in the spike protein. Right: Detailed AggreScan^64^ analysis of aggregation in the FPR1 and neutrophil activators. Percentages indicate the aggregation positive fraction. See also Table S3. (E) Left: AggreScan aggregation hotspots of the known FPR1 activators T20 from HIV-1 and gG-2p20 from HSV-2. Right: Aggregation hotspots can also be found in the envelope protein GP350 of HHV4, the envelope glycoprotein E of VZV, glycoprotein K8.1 from HHV8, Flury glycoprotein of rabies virus. (F) Calcium mobilization of the envelope proteins of viruses listed in (E) to FPR1-, FPR2-, FPR3-or Mock-transfected HEK293T cells (*n* = 3). Data are represented as mean ± SD. Statistics were performed using Dunett's post-hoc one-way ANOVA to a reference that is indicated by #. Significances are ∗*p* ≤ 0.05; ∗∗*p* ≤ 0.01; ∗∗∗*p* ≤ 0.001. See also Figure S7.

The localization of FPR activators in the 3D-structure of the SARS-CoV-2 omicron B.1.1.529 spike protein indicates that most interaction sites for FPR1, FPR2, and FPR3 are centered around the tip of the spike protein that contains the receptor-binding domain (Figure 5A). A comparison with published datasets revealed the presence of IgG antibodies that cover the near complete sequence of several FPR ligands in the serum of patients with COVID-19 (Figure 5B). Moreover, corresponding peptide fragments were also found in nasopharyngeal swab samples.^55^^,^^56^^,^^57^^,^^58^^,^^59^^,^^60^^,^^61^^,^^62^^,^^63^ Fragments of 4 of the identified FPR1 or FPR2 activators, as well as fragments of 9 FPR3 activators could be verified via mass spectrometry^55^^,^^56^^,^^59^^,^^60^^,^^61^^,^^63^ (Figure 5B; Table S2). Furthermore, IgG antibody epitopes spanning 3 individual FPR1/2-dependent neutrophil interaction regions and some FPR3 activators were revealed in high-density peptide microarrays.^57^^,^^58^^,^^62^ Two of the epitopes belong to the most immunoreactive spike protein epitopes, whose IgG antibodies are present in at least one-fourth of the patients. Their frequency even exceeded the immunoreactivity of receptor-binding domain-targeting antibodies.^58^ One of the two epitopes corresponds to parts of the neutrophil/FPR1 activator region GCLIGAEYVNNSYEC near the furin cleavage site. Interestingly, the other epitope covers the MPR region EQYIKWPWYIWL, which clearly indicates that MPR peptides interact with the immune system. All identified IgG epitopes were significantly enriched in patients with severe COVID-19. Farrera-Soler et al. reported the epitope presence in >40% of the patients, Acharjee et al. in ≥25% and Mishra et al. in even up to 69%. In addition, Acharjee et al. and Mishra et al. showed that severity in COVID-19 was linked with high amounts of antibodies against these respective IgG epitopes.

The distribution of activators for the individual FPR subtypes displays clear differences. FPR1 seems to be focused on the spike protein tip, whereas FPR2 and FPR3 also recognize additional sites that are localized in domains that connect the S1 subunit toward the S2 subunit. In order to better understand the peptide properties that lead to the differential FPR activation pattern, we aimed to analyze their physiochemical properties in closer detail. While the overall spike protein displayed hydrophilicity, we observed that the FPR1 interaction sites were far more hydrophobic than the average spike protein sequence (Figure 5C). Given that these domains are located at the surface of the spike protein that is in close contact with the aqueous environment, this is an unusual result that indicates the use of these sites for protein-protein interactions. We used the AggreScan tool that permits the prediction of protein aggregation sites to test this hypothesis.^64^ The analysis revealed a multitude of potential aggregation hotspots in the SARS-CoV-2 spike protein (Figure 5D). Consistent with the idea that FPR1 might focus on the detection of protein-protein interaction sites, we observed a clear enrichment of aggregation hotspots in FPR1 activators. 71,6% of the peptide sequences of FPR1 activators were aggregation positive, whereas their mean frequency of aggregation hotspots in the whole peptide pool is only 45.5%. Of note, we also found that 90% (9/10) of FPR1 activator peptides, as well as 92,3% (12/13) of neutrophil activators did contain aggregation hotspots. However, not all aggregation hotspot containing peptides are FPR activators because 40 of the 65 of the peptides that did neither activated FPR1 nor neutrophils also contain aggregation hotspots (Table S3). Our data suggests that the length of aggregation hotspots might be critical because in peptides that were activators of FPR1 or neutrophils the aggregation hotspot length was on average 9,7 and 9,4 amino acids, respectively. In contrast, the length of the aggregation hotspots in non-activators was, on average, only 6,8 residues.

Interestingly, similar aggregation hotspots exist in the viral FPR1 agonists T20^33^ that is present in the gp41 protein of HIV-1, and the gG-2p20 peptide^65^ from Herpes simplex virus 2 (HSV-2) glycoprotein G (Figure 5E). We thus wondered if surface proteins from other viruses may also contain aggregation hotspots that might serve as FPR1 activators. A subsequent analysis of the envelope glycoprotein gp350 from Epstein-Barr virus (EBV) HHV4, the envelope glycoprotein E from Varicella zoster virus (VZV), glycoprotein K8.1 of the Human herpesvirus HHV8 and the HEP-Flury glycoprotein from the Rabies virus with the AggreScan tool revealed that the sequence of these envelope proteins contains 22.1%, 35.2%, 27,6% and 31,3% aggregation hotspots, respectively. To test if they can also activate FPRs, we used peptide pools of these viral surface proteins and tested them for their activity on transfected HEK293T cells. As expected, all tested pools could activate FPR1, FPR2, and FPR3 (Figures 5F and S7). FPR1 activation started at 10 to 100 nM concentrations. Compared to FPR1, the FPR2 responses were clearly less sensitive and displayed a lower signal amplitude. However, FPR3, which is rarely activated by any known FPR1 or FPR2 agonist, displayed responses that were similar or even exceeded those of FPR1 in terms of affinity and signal intensity. Together, this suggests that FPR1 interaction partners that are based upon aggregation-prone protein sites exist in many further viral proteins of various unrelated viral strains. In addition, these results suggest that FPRs and particularly FPR3 may play a previously unnoticed role in many different types of viral infections through an interaction with envelope protein fragments from miscellaneous virus.

## Discussion

Formyl peptide receptors are a family of immune receptors that are expressed in various parts of the body^1^^,^^10^^,^^11^^,^^66^ where they help to regulate a considerable number of important immune functions. We here significantly expand the number of viral FPR ligands. Furthermore, our data raise multiple independent lines of evidence for a yet unappreciated general relevance of FPRs for viral infections. We observed a differential upregulation of the individual FPR subtypes during COVID-19 infections in several different infection-relevant cell types in the human lung. This study is also the first to report that many spike ectodomain peptides, of which fragments and IgG epitopes are found in patients with COVID-19, are able to activate innate immune cells, such as primary isolated human neutrophils, in an FPR-dependent manner. Next, we can show that multiple peptides from envelope proteins of different viruses can interact with FPRs and that these peptides trigger distinct signal transduction pathways in innate immune cells, which in turn activate important immune responses. Moreover, we can show that these responses can be strongly attenuated through MPR peptides that are present in a number of completely different virus types. Thus, MPR peptides from different viral strains can potentially be exploited for a targeted modulation of different immune functions. Finally, we identified a surprisingly large number of FPR3 activators, which argues for an unrecognized role of FPR3 during viral infections. Given the broad distribution of FPRs in different immune-relevant cell types and the multifaceted role of FPRs in immune responses, this newly discovered set of interaction partners potentially affects numerous physiological processes.

The precise elucidation of the individual biological role of each FPR subtype during different types of viral infection will likely constitute a complex challenge, as our data show that many viral peptides interact with more than one receptor (Figures 1, 2, 4, S2, and S5). Published research has already demonstrated that the biological role of FPRs can be quite divergent in different viral infections. For example, in influenza A virus infections AnxA1, which is incorporated into the virus envelope during the budding process, acts as a crucial interaction partner for FPR2.^23^ This promotes an ERK-dependent increase in viral replication and the release of cytokines such as IL-6 and IFN-β.^23^ The application of FPR2 antagonists such as WRW_4_, PBP10, and t-Boc2 or pretreatment with a monoclonal mFpr2 antibody during influenza A infection diminished viral loads and increased survival rates in infected mice.^23^^,^^24^^,^^25^ By contrast, AnxA1/FPR2 interaction plays a protective role in dengue virus infections^27^ because there the absence of either FPR2 or AnxA1 in mice led to disease deterioration and exacerbated inflammation, whereas the application of the FPR2 agonist Ac_2-26_ that corresponds to the N-terminal AnxA1 domain, alleviated disease severity.^27^ In HIV-1 infection, FPR2 on the one hand may inhibit viral infection through HIV-1 co-receptor CCR5 and CXCR4 cross-desensitization mediated by activation through glycoprotein fragments,^28^^,^^29^^,^^33^^,^^67^ but on the other hand can act as a co-receptor for HIV-1 viral entry itself.^68^ Thus, the exact role of FPR2 during HIV-1 infections remains elusive.^5^

FPRs can trigger complex cascades of pro- or anti-inflammatory signaling,^16^^,^^20^ and there is accumulating evidence for ligand-dependent biased signaling.^20^^,^^69^^,^^70^ For example, it is well known that the activation of FPR1 usually results in pro-inflammatory signaling,^1^^,^^2^^,^^5^ whereas in the case of FPR2, the resulting cellular effects are not as clear, as both pro- and anti-inflammatory signaling have been reported.^7^^,^^12^^,^^16^^,^^20^ A recent study that compares different signaling pathways of FPR1 and FPR2 demonstrates that different FPR agonists that act on the same receptor can produce distinct signal transduction profiles.^71^ Moreover, a given activator can frequently interact with more than one receptor.^1^^,^^71^ Interestingly, our data demonstrate that the resulting responses to viral peptides can depend on the precise amino acid composition and length (Figures S3 and 3). Thus, it is conceivable that different virus variants can exploit FPR signaling to modulate innate immune responses in a virus subtype specific manner. This could be a key strategy for the development of new virus variants to successfully reinfect a host. Testing this hypothesis will be technically challenging because FPR signal transduction can vary between different cell types, and FPRs are expressed in a number of intertwined immune cell types. Thus, a complete analysis of the precise signaling events of a given virus variant in several cell types will be required.^1^^,^^2^

Besides viral peptides that interact with FPR1 and FPR2, we identified a large novel group of FPR3 specific viral activators - a receptor for which very few biological relevant activators have been described yet,^53^^,^^72^ leaving much room for speculations about its major biological function.^1^^,^^2^ In contrast to FPR1 and FPR2, for which a large panel of receptor subtype-selective ligands have been described, only two biological relevant agonists with a known preference for FPR3 were identified. One is the mitochondrial-derived neuroprotective peptide Humanin^51^^,^^52^^,^^73^ that binds to FPR3 and FPR2 with high affinity^51^^,^^52^ and was recently shown to be attenuated in the serum of patients with COVID-19.^73^ The second one is F2L, an acetylated 21mer N-terminal cleavage product that activates FPR3 at low nanomolar and FPR2 at low micromolar concentrations, which is released from the heme-binding-protein-1 N-terminus by cathepsin D.^53^ Interestingly, hemolysis-derived heme has been shown to trigger a variety of fatal proinflammatory effects during COVID-19^74^ and several SARS-CoV-2 proteins have been shown to bind to hemoglobin and its metabolites.^75^ Notably, FPR3 is predominately expressed in plasmacytoid dendritic cells, eosinophils, and several types of tissue-specific macrophages that are known to play a crucial role in antiviral immunity.^76^ Thus, there is at least some circumstantial evidence that points toward a possible role of this receptor in viral infections. The identification of at least five FPR3-selective peptides (Figure S6) will help to better understand the role of this so far poorly characterized receptor.

To date, we do not know much yet about the precise release mechanisms and local concentrations of the viral peptides capable of interacting with FPRs. However, it is reasonable to assume that viral envelope proteins will be subjected to multiple different types of protease digestions.^46^^,^^47^^,^^48^ This would argue for a considerable number of different spike protein peptide fragments of variable length and amino acid structure that can be found at sites of infection. This hypothesis is supported by the findings that numerous different spike protein-derived peptide fragments have been verified in nasopharyngeal swabs samples of patients with COVID-19 by mass spectrometry.^55^^,^^56^^,^^59^^,^^60^^,^^61^^,^^63^ Notably, many of these fragments match well with the peptide sequences of the FPR1-3 and neutrophil activators that we identified in this article (Figure 5B, Table S2). However, it is common practice to perform protease digestions using proteases such as trypsin before mass spectrometry analysis. Thus, we cannot be sure whether the fragments observed in patient samples cover the full length of our activators and what type of degradation process have led to their release. One possible release mechanism might be neutrophil elastase cleavage of the spike protein, as this protease occurs in many viral infections and was linked to FPR activity in patients with COVID-19.^48^ An *in-silico* analysis of the spike protein sequence for neutrophil elastase cleavage sites revealed that several of the predicted fragments would contain major parts of the peptide regions that we identified as FPR-dependent neutrophil activator domains (Figure S8). We also found supportive evidence that the FPR ligand domains within the spike protein can trigger an adaptive immune response, because we found many major immunoreactive IgG epitopes from COVID-19 patient serum. They also include large parts of the MPR domain as well as other FPR activating regions (Figure 5B, Table S2).^57^^,^^58^^,^^62^ Notably, immunoreactivity of these epitopes was present in considerable 25–69% of patients with severe COVID-19.^57^^,^^58^^,^^62^ These epitopes seemed to be exclusively present in patients with symptomatic SARS-2 as they are not present in patients infected with SARS-CoV-1, other HCoVs, or healthy controls.^57^ Moreover, certain epitopes displayed high immunoreactivity in addition to their frequent occurrence.^58^ Interestingly, epitopes from HIV that are similar to this region have also been described as targets to produce neutralizing HIV-1 antibodies.^37^^,^^77^^,^^78^ Taken together, this strongly suggests that the MPR domain and the other activators described here or similar fragments are released during infections and can therefore participate in the modulation of local immune cells. We could also show that some of the MPR peptides from different viral strains not only abrogate FPR1-dependent calcium flux in neutrophils but can also differentially modulate several other important innate immune functions, such as migration and MMP-9 protease release. Hence, the precise biological outcome may change depending on the viral strain. Lastly, the viral envelope protein fragments will have to compete with various other host endogenous FPR ligands such as annexin A1, resolvin D2, lipoxin A4, and formylated peptides from damaged mitochondria for binding to the FPRs.^1^^,^^48^ Thus, one may expect highly dynamic alterations in FPR signaling that depend on the stage of infection.

The exact molecular recognition mechanisms that permit FPRs a specific interaction with such a broad spectrum of structurally diverse ligands still pose a fascinating molecular riddle. Our data provide new insights into the rationale of the underlying exceptional detection mechanism^1^^,^^2^ that permits a broad, but highly specific sensing of structurally diverse formylated bacterial signal peptide fragments for the defense against bacteria.^6^^,^^18^ This mechanism is already known to also contribute to a number of unwanted pathophysiological processes.^1^^,^^79^ A prominent example is Alzheimer’s disease, where FPRs have recently been shown to interact with different variants of amyloid beta,^14^^,^^16^^,^^80^ serum amyloid A,^81^ and prion protein,^15^ that are well known for their capability to form peptide aggregates. Several cryo-electron microscopy studies have analyzed the binding of several ligands to FPR1 and FPR2, but *in silico* tools for the prediction of FPR ligands are still missing.^82^^,^^83^^,^^84^^,^^85^ During the course of this study, we used the AggreScan algorithm to identify novel FPR activators in other viral proteins. The AggreScan^64^ tool, which was developed to determine aggregation hotspots in amyloid beta and other aggregation prone proteins proved to be a useful tool for the *in silico* identification of potential FPR interaction partners. Our analysis of the spike protein sequence provided evidence that FPR1 interacting spike protein fragments also contain aggregation hotspots. Furthermore, we can show that similar aggregation hotspots are also present in other published FPR1 peptides from unrelated viruses, such as the T20 from HIV-1^33^ and gG-2p20 from HSV-2.^65^^,^^86^ Taken together, these observations argue that an analysis of aggregation hotspots in proteins can be a useful strategy to identify yet undiscovered ligands for FPRs. However, the results on FPR1 and the other receptors show that additional, yet not clearly identified structural features play a role for the interaction with the individual FPR subtypes. Our previous studies^18^^,^^21^^,^^54^ indicate that the ligands may need a specific 3D conformation within the receptor binding site. Recent progress in solving the receptor structure^82^^,^^83^^,^^84^^,^^85^ and ligand prediction^87^ will be extremely helpful to solve this puzzle. Thus, our discovery that the FPR detection mechanisms is prone to the detection of aggregation hotspots can explain some of the known interactions with viral, bacterial, and host endogenous peptides. A better understanding of the broad detection capabilities of FPRs may pave the way toward a systematic *in silico* prediction of FPR ligands, which can lead to the discovery of novel pathophysiological processes that involve FPRs.

### Limitations of the study

This study mainly uses *in vitro* data with the aim to understand the molecular mechanism, however besides indications through publicly available meta data, we can only estimate about the effects *in vivo*. Also, we vastly utilized synthetic peptides of the viral areas of interest, but have not worked with viruses, primary isolates, or viral infection supernatants.

## Resource availability

### Lead contact

Requests for further information and resources should be directed to and will be fulfilled by the lead contact, Bernd Bufe (bernd.bufe@hs-kl.de).

### Materials availability

All relevant materials are available from the lead contact upon request.

### Data and code availability

All data in this article are available upon request. To request access, please contact Bernd Bufe (bernd.bufe@hs-kl.de) as the lead contact. This article does not report original code. This study did not generate any unique reagents. Any additional information required to reanalyze the data reported in this article is available from the lead contact upon request. This article analyzes existing, publicly available data, accessible at CELLxGENE or NCBI (see STAR Methods section).

## Acknowledgments

We would like to thank Markus Bischoff for providing helpful feedback and comments on the article. This work was kindly funded by the Ministry of Science and Health Rhineland-Palatinate (MWG) through the HWA-Corona-KI project and the Research Colleges MultiSensE and NeurodegX. Further funding was provided by the 10.13039/501100002347Federal Ministry of Education and Research (BMBF) for the PepSensE project (13FH521KX9) and the 10.13039/501100001659German Research Foundation (DFG; Lo 1853/1-2 and INST 252/19-1 FUGG) and intramural funding by the Saarland University (Young Investigator Grant).

## Author contributions

Experimental procedures: H.H., L.B., C.B., I.M., and A.T.; data analysis: H.H., L.B., B.B., and S.J.; conceptualizing: H.H., B.B., L.B., and S.L.; article drafting: H.H. and B.B.; supervision: B.B., S.L., and L.B.; funding: B.B. and S.L.; ethics application: S.L.; contributions according to stated order. All authors approved the article.

## Declaration of interests

The authors declare no competing interests.

## STAR★Methods

### Key resources table

**Table:** 

REAGENT or RESOURCE	SOURCE	IDENTIFIER
**Antibodies**		

Phospho-ERK1/2 Thr202/Tyr204	BioLegend	Cat# 369501; RRID: AB_2721734
HRP Goat anti-mouse IgG	BioLegend	Cat# 405306; RRID: AB_315009
HRP conjugated β-Actin	Proteintech GmbH	Cat# HPR-6609

**Bacterial and virus strains**		

Competent bacteria	Promega	Cat#L1195

**Biological samples**		

Isolated human polymorphonuclear granulocytes (PMN)	Institute of Virology, Saarland University Medical Center	NA

**Chemicals, peptides, and recombinant proteins**		

Detailed list of peptides is supplied as Table S1	NA	NA
Dulbecco’s Modified Eagle’s medium	Biowest	Cat#L0106-500
Heat-inactivated fetal calf serum (FBS Good)	PAN Biotech	Cat#P30-19375
Penicillin-streptomycin	Biowest	Cat#L0022-100
L-glutamine	Biowest	Cat#X0551-100
RPMI-1640	Biowest	Cat#L0501-500
Dimethyl sulfoxide	Sigma-Aldrich	Cat#D4540
Poly-D-lysine	Sigma-Aldrich	Cat#P6407
Dulbecco’s Phosphate Buffered Saline	Biowest	Cat#L0615-500
Ca2+/Mg2+-free Dulbecco’s PBS	PAN Biotech	Cat#P04-36500
Pancoll Human 1.077 g/mL	PAN Biotech	Cat#P04-60100
Hypotonic dH2O (aqua H2O for injection)	B BRAUN	Cat#L8501-01
jetPEI transfection reagent	Polyplus	Cat#101000020
Calbryte 520 AM	AAT Bioquest	Cat#20653
NaCl	Carl Roth	Cat#HN00.2
HEPES	Carl Roth	Cat#HN78.2
KCl	Carl Roth	Cat#781.3
CaCl2	Carl Roth	Cat#HN04.2
Glucose	Carl Roth	Cat#6780.1
Normal Frog Ringer	EcoCyte BioScience	Cat#LRE-S-LSG-1004-7
Hoechst 33342	Thermofisher	Cat#62249
Coelenterazine	Carl Roth	Cat#4094.4
Incucyte Cytotox Dye	Sartorius	Cat#4632
PBS (ROTIFair 7.4)	Carl Roth	Cat#1112.2
ROTI®Nanoquant	Carl Roth	Cat#K880.1
LDS sample buffer	GenScript	Cat#M00676
NuPAGE reduction agent	Invitrogen	Cat#NP0009
10% Bis-Tris gel	Genscript	Cat#M00666
Ponceau S	Carl Roth	Cat#5938
Tween20	Carl Roth	Cat#9127
2xYT broth	Carl Roth	Cat#X966.1
Tris-HCl	Carl Roth	Cat#9090.3
EDTA	Sigma-Aldrich	Cat#E5134
Triton-X	Sigma-Aldrich	Cat#X100
DTT	Carl Roth	Cat#6908.1
Complete protease inhibitor	Roche	Cat#11836153001

**Critical commercial assays**		

Human Active MMP-9 Fluorokine E Kit	R&D systems	Cat#F9M00
ECL Prime Kit	GE Healthcare	Cat#28980926
PureYield Plasmid Midiprep System	Promega	Cat#A2495

**Experimental models: Cell lines**		

HEK293T	ATCC	Cat#CRL-1573
HL-60	CLS	Cat#300209

**Recombinant DNA**		

G-protein subunit G_α16_ encoding plasmid	Bufe et al., 2015^18^	NA
pcDNA3.1 vector	Invitrogen	Cat#11510866
Human FPR1 gene in pcDNA3.1 vector	Bufe et al., 2015^18^	NA
Human FPR2 gene in pcDNA3.1 vector	Bufe et al., 2015^18^	NA
Human FPR3 gene in pcDNA3.1 vector	Bufe et al., 2015^18^	NA
Plasmid encoding a membrane-bound Gaussia Luciferase (GLuc-PM) biosensor^88^	Laszlo Hunyady	Addgene plasmid #164783

**Software and algorithms**		

MetaXpress software	Molecular Devices	NA
ProtParam	expasy.org	NA
AggreScan	bioinf.uab.es/aggrescan	Conchillo-Solé et al.^64^
PeptideCutter	expasy.org	NA
BioRender	biorender.com	NA
GraphPad Prism	graphpad.com	NA
ChimeraX-1.6.1	cgl.ucsf.edu/chimerax/	NA

**Other**		

Black optical 96-well microplates	μclear, Greiner Bio-One; or Phenoplates, PerkinElmer	Cat#655090; Cat#6055302
S-Monovettes (Citrate, 10 mL 9NC tubes)	Sarstedt	Cat#02.1067.001
cellQART 24-Well Insert 3.0 μm PET translucent	Sabeu	Cat#9323002
24-well plates	Falcon	Cat#353047
Protein low bind tubes	Eppendorf	Cat#0030108116
Wet Tank blotting system	BioRad	NA
ImageXpress Micro high-throughput confocal microscope	Molecular Devices	NA
Fluorescence imaging plate reader system (FLIPR tetra)	Molecular Devices	NA
IncuCyte S3 system	Sartorius	NA
FLEXSTATION III multimode reader	Molecular Devices	NA
10M multi-mode plate reader	Tecan Spark	NA
Microplate washer (ELx50)	BioTek	NA
LumiImager	Roche	NA

### Experimental model and study participant details

#### Cell culture of HEK293T and HL-60 cell lines

All cells were consistently incubated at 37°C and 5% CO_2_. HEK293T cells were cultivated in Dulbecco’s Modified Eagle’s medium supplemented with 10% (v/v) heat-inactivated fetal calf serum, 1 unit/ml penicillin-streptomycin and 2 mM L-glutamine. HEK293T cells were passaged two times per week and kept below 90% confluence. HL-60 cells were cultivated in equally supplemented RPMI-1640 and were passaged two times per week to a cell density of 0,3∗10^6^/mL. HL-60 differentiation into a neutrophil-like state was achieved by adding 1,3% (v/v) dimethyl sulfoxide for an incubation period of six days. Only differentiated HL-60 cells (dHL-60) were used for experiments. Subsequent procedures and experiments were performed on black optical 96-well microplates coated with 10 μg/mL poly-D-lysine in Dulbecco’s Phosphate Buffered Saline. Cells were seeded onto these plates to the following densities: HEK293T 20–30% confluence; dHL-60 2,5-3∗10^5^/well.

#### Isolated human polymorphonuclear granulocytes (PMN; neutrophils)

This study used blood from healthy adult volunteers that was collected for the isolation of polymorphonuclear granulocytes (PMN; neutrophils). The cells were isolated just prior to the experimental procedures, were resuspended in RPMI-1640 and intermediately stored on ice up until their use. The sample size (n) for each collected dataset with neutrophils likewise represents the number of donors for the given experiment; hence neutrophils of each donor accounted for one biological repetition. The donors were randomly and without the knowledge of the experimenter chosen and allocated to the assays. The local Ethics Review Committee (Ärztekammer des Saarlandes, Saarbrücken, Germany) approved the scientific use of the human blood samples in this study. All experiments were performed according to the relevant guidelines and regulations including the Declaration of Helsinki. Written informed consent was obtained from all donors. The influence of sex, gender, or both on the results of the study has not been evaluated.

### Method details

#### Isolation of human polymorphonuclear granulocytes (PMN; neutrophils)

Initially, 10 mL of blood from healthy adult volunteers were collected into S-Monovettes (Citrate, 10 mL 9NC tubes). Subsequently, 10 mL of Ca^2+^/Mg^2+^-free Dulbecco’s PBS was added. The cell suspension was carefully overlaid onto 12 mL of Pancoll (Pancoll Human 1.077 g/mL) and centrifuged at 800 x g for 30 min at room temperature. After aspiration of plasma, PBMCs and Ficoll layers, the granulocyte-containing phase (approx. 5 mL) was transferred to a 50 mL Falcon tube. The lysis of the erythrocytes was induced by adding cold hypotonic dH_2_O (aqua H2O for injection) until the 45 mL mark. After 30 s of erythrocyte lysis, 5 mL sterile 10 x PBS was added and mixed, thereby stopping the lysis. After centrifugation for 5 min at 1500 rpm the supernatant was removed, and the lysis procedure was repeated. The pellet was gently resuspended in RPMI-1640 supplemented with 1% penicillin-streptomycin, 1% 100 mM sodium pyruvate, and 10% FCS. Cell purity was consistently >95% CD66b positive in fluorescence-activated cell sorting (FACS).

The isolated polymorphonuclear granulocytes were stored on ice until their use in experiments. To prepare for experimental procedures, PMNs were centrifuged at 300 x g for 10 min, resuspended in the appropriate buffer (ringer solution) or media (RPMI-1640) and were subsequently seeded onto PDL-coated black optical 96-half-well μCLEAR-plates microplates to a density of 2.5∗10^5^/well (calcium imaging) or 6∗10^4^/well (NETosis). Throughout this work, the cell type nomenclature of PMNs and neutrophils are used synonymously.

#### HEK293T transfection

At a confluence of 50–70% (approx. 24h after seeding), HEK293T cells were transiently transfected using jetPEI transfection reagent according to manufacturer’s protocol. Here, 0.125 μg of receptor coding pcDNA3.1 plasmid and 0.125 μg of a G-protein subunit G_α16_ encoding plasmid were added to each well.^14^^,^^18^^,^^54^ Receptor plasmids were substituted by an empty pcDNA3.1 vector for mock negative control transfections. Cell supernatant of transfected HEK293T cells was replaced by fresh cell culture medium 24h after transfection. Experiments with transfected HEK293T cells were eventually ruled out at 48h after transfection.

#### Calcium imaging

Calcium imaging was performed as a high-throughput readout for cell population activation using a fluorescence imaging plate reader system (FLIPR tetra).^18^^,^^54^ Therefore, cells seeded in 96-well plates were incubated with calcium-sensitive dye Calbryte 520 AM (2 μM) in their respective buffer at room temperature. HEK293T cells were loaded for 2 h in physiological C1 buffer (130 mM NaCl, 10 mM HEPES, 5 mM KCl, 2 mM CaCl_2_, 5 mM glucose, pH 7.4), dHL-60 and PMNs were loaded in premixed ringer solution (90 mM NaCl, 5 mM HEPES, 2 mM KCl, 2 mM CaCl_2_, 1 mM MgCl_2_, 5 mM glucose) for 1 h. dHL-60 and PMNs were taken from suspension culture just prior to the experiments, were centrifuged at 300 x g for 10 min, subsequently resuspended in the appropriate amount loading solution and directly seeded into 96-well plates (as described above). After the incubation time with the calcium-sensitive dye, cells were rinsed with their respective buffer for three times using a microplate washer and were left to rest for 10 additional minutes before starting the calcium imaging process. Acquisition of baseline fluorescence (F_0_) was performed for 25 s before ligand application. Calcium responses were calculated as dF/F_0_ values (signal amplitude dF divided by mean baseline fluorescence F_0_).

#### Chemotaxis assays

Cell migration of neutrophils was monitored by utilizing a Boyden Chamber Technique. For that, we used cellQART 3.0 μm PET translucent inserts in 24-well plates. The respective cells were centrifuged at 300 x g for 10 min and resuspended in RPMI-1640 containing 0.5% (v/v) FCS, 1 unit/ml penicillin-streptomycin and 2 mM L-glutamine. 2∗10^5^ cells were placed into the top chamber of each well in a volume of 200 μL. Afterward, cells were left to settle for 30–60 min at room temperature. For inhibitor experiments, inhibitors were added to the cells in the top chamber during that time. After the settling time diluted ligands (with or without additional inhibitors) were placed into the bottom chamber. Cell migration was carried out over a time span of 90 min during which the cells were incubation at 37°C and 5% CO_2_. Eventually, cell inserts were removed and image acquisition of 36 side-by-side sites per well of migrated cells at the plate bottom was performed using an incubated IncuCyte S3 system. Migration was assessed via cell confluency and normalized to positive control response to compensate variability between donors.

#### Binding assays

Binding assays were prepared by incubating FPR1-, FPR2-, FPR3-or Mock-transfected HEK293T cells with fluorescently labeled peptides together with 20 μM of Hoechst 33342 nuclear dye in fully supplemented DMEM cell culture media (10% (v/v) heat-inactivated fetal calf serum, 1 unit/ml penicillin-streptomycin and 2 mM L-glutamine) for 30 min at 37°C and 5% CO_2_. Afterwards, cells were rinsed at least ten times with C1 buffer (130 mM NaCl, 10 mM HEPES, 5 mM KCl, 2 mM CaCl_2_, 5 mM glucose, pH 7.4) just prior to image acquisition. Image acquisition was performed using an ImageXpress Micro high-throughput confocal microscope with acquisition of three sites per well. Binding analysis was performed using a cell scoring module in the built-in MetaXpress software. For each site per well, the positive cell fraction was normalized to the total number of cells and the largest datapoint of the respective dataset.

#### Bioresonance energy transfer assay

Bioresonance energy transfer assay (BRET) was prepared by co-transfection of HEK293T cells with 0.5 ng of plasmid encoding a membrane-bound Gaussia Luciferase (GLuc) biosensor^88^ together with 0.125 μg of FPR1-or Mock-plasmid. BRET assays were performed based on a protocol by Tóth et al.^88^ measuring the displacement of a fluorescently labeled ligand through application of coronavirus MPR peptides. The BRET ratio represents the ratio of acceptor fluorescence and donor bioluminescence emission. For FPR1 transfected cells, a fluorescence-coupled FPR1 binding peptide served as energy acceptor. Pre-warmed ligands (MPR peptide and the fluorescent probe; 30°C) were applied to cells in supplemented DMEM cell culture media, incubated together at 37°C for 30 min and subsequently rinsed 3x with physiological C1 buffer. GLuc substrate coelenterazine (1 μM) was added, immediately followed by acquisition of light emission of the ligand-bound TAMRA dye at 580–700 nm and the gaussian luciferase at 475–500 nm, respectively in a multi-mode plate reader. Changes in BRET ratios were determined by comparisons with fluorescence-coupled probe in the absence of MPR peptides. Average basal BRET ratios measured in Mock/GLuc co-transfected HEK293T cells were subtracted from total BRET ratios as a background correction due to the overlap of donor emission at the acceptor acquisition wavelength.^89^

#### NETosis assay

For NETosis measurement, isolated PMNs were centrifuged at 300 x g for 10 min and then resuspended in RPMI-1640 containing 0.5% (v/v) FCS, 1 unit/ml penicillin-streptomycin and 2 mM L-glutamine and 125 nM of Incucyte Cytotox Dye. After a settling time of 30 min at room temperature, ligands were added to their final concentration. After the settling time, cells were transferred into the incubated IncuCyte imaging system at 37°C and 5% CO_2_. NETosis after 4h was calculated via IncuCyte software tools as dye-stained area normalized to cell confluency as well as the largest value of the given dataset to compensate variability between donors. Regular dead cells were excluded from the analysis.

#### MMP-9 assay

MMP-9 activity was measured using the commercially available R&D systems Human Active MMP-9 Fluorokine E Kit by following the manufacturer’s instructions. Briefly, 5x10^6^ isolated PMNs were centrifuged (300 x g; 10 min), washed with PBS (ROTIFair 7.4) and resuspended in 250 μL ringer solution containing the respective ligands. Stimulation was performed for 15 min at room temperature. Thereafter, the cells were centrifuged (300 x g; 10 min), supernatant was collected and diluted 100-fold with the calibrator diluent RD5-24 included in the kit. 200 μL of this dilution served as input for the activity assay.

#### ERK1/2 phosphorylation assays

300.000 cells were seeded in each well of an uncoated 12-well plate. After 30 min settling time on ice, cells were treated with the respective stimulants and incubated at 37°C, 5% CO_2_ for 15 min. Cells were subsequently washed with 500 μL PBS and then lysed with 250 μL protein extraction buffer (50 mM Tris-HCl pH 8.0, 5 mM EDTA, 150 mM NaCl, 0.5% [v/v] Triton-X, 5 mM DTT, Roche complete protease inhibitor) for 5 min on ice. Lysates were then placed into protein low bind tubes (Eppendorf) and treated for 20 min in an ultrasonic bath. Samples were centrifuged at 4°C, 12.000 rpm for 20 min to remove debris. Supernatants were collected and stored at −20°C until further use. Protein concentrations were determined using ROTINanoquant in a FLEXSTATION III multimode reader. Samples were mixed with LDS sample buffer and NuPAGE reduction agent and subsequently heated at 70°C for 10 min 20 μg of each sample were loaded onto a 10% Bis-Tris gel. SDS-PAGE was performed at 120 mV for 40 min. Proteins were blotted onto nitrocellulose membranes using a Wet Tank blotting system at 350 mA for 60 min. Transfer was controlled by Ponceau S staining. Membranes were then blocked with 3% BSA and 0.1% Tween 20 in PBS (PBST) for 60 min. Primary antibody against pERK1/2 (Phospho-ERK1/2 Thr202/Tyr204) was diluted in this blocking buffer to 0.5 μg/mL and incubated overnight at 4°C on an orbital shaker. Membranes were then washed three times for 10 min each with PBST and subsequently treated with 0.1 μg/mL secondary antibody (HRP Goat anti-mouse IgG) in blocking buffer for 60 min. Membranes were again washed three times for 10 min each with PBST. Detection of luminescence signals was performed with ECL Prime Kit in a LumiImager. Membranes were stained afterwards with 2.5 μg/mL actin control (HRP conjugated β-Actin) in blocking buffer for 1h at RT. Signals of actin staining were measured as described above. Raw signals of proteins of interest were normalized to respective actin control.

#### Analysis of FPR expression in COVID-19 and SARS-CoV patients

Gene expression of FPR1-3 and housekeeping genes in SARS-CoV-2 patients was analyzed using the publicly available CELLxGENE database.^42^ From this database, all 24 at the time available source datasets (data gathered 12-20-2023) were accessed by filtering for tissue type (lung) and disease type (COVID-19 and normal). The summarized data of these publications was taken into account for analysis. As many specialized cell subsets however lack sufficient data, we excluded cell types that had less than 50 *FPR1 gene* expressing cells to focus only on statistically adequate and reliable data. *FPR1 gene* expression was chosen as a filter because it constitutes the gene of interest that is found in most included lung cell types. Data are displayed as scaled mean normalized expression values.

SARS-CoV patient PBMC gene expression of FPR1-3 was retrieved from NCBI GEO Dataset (accession: GDS1028, ID: 1028; GEO Profile: FPR1 ID: 9264589, FPR2 ID: 9267790, FPR3 ID: 9268454).

#### Hydrophobicity, aggregation hotspots and neutrophil elastase cleavage sites

Peptide sequence hydrophobicity was calculated as the grand average of hydropathicity index (GRAVY) by SwissProt/Expasy tool ProtParam. Aggregation hotspots were calculated using the publicly available online tool AggreScan.^64^ Neutrophil elastase cleavage sites were identified using SwissProt/Expasy tool PeptideCutter. Representative images were created using BioRender online tool and exported under a paid subscription.

#### Ligands

Peptide stocks were stored at −20°C. Approximately 1h prior to functional experiments, peptides were thawed at room temperature. Dilution to the final concentration was achieved in the physiological buffer C1 assay buffer or ringer solution. Detailed information for each peptide is provided in Table S1.

#### Cloning of human FPR genes

FPR1, FPR2 and FPR3 were amplified from human genomic DNA and cloned into pcDNA3.1^(+)^ vector as previously described.^18^ Plasmids were introduced into competent bacteria, amplified overnight (rotary incubator, 150 rpm; 2xYT broth), prepared using the PureYield Plasmid Midiprep System according to manufacturer’s protocol and purified in an isopropanol precipitation. Lastly, plasmid concentration was adjusted to 1 μg/μL. Plasmids were stored at −20°C. FPR coding gene sequences of this study are published in the supplement information of Bufe et al., 2015.^18^

#### Peptide and protein models

Peptide models were generated by the ChimeraX-1.6.1 build in structure editing tool. Protein structures were accessed via the annotated PDB accession numbers. Surface was colorized individually.

### Quantification and statistical analysis

#### Graphs and statistics

Statistical calculations were performed using GraphPad Prism software under paid license. Statistical significance was calculated using either one-/two-way ANOVA with Dunnett’s/Šídák’s multiple comparisons post hoc analysis, the unpaired student’s t-test with assumption of Gaussian distribution or the Wilcoxon-Mann-Whitney-Test. The used tests as well as the *n* values are denoted in the figure legends. Probability of error alpha values were settled as 0.05. Statistical tests were two-sided. Graphs display mean values ± standard deviation. Significances are displayed as ∗*p* ≤ 0.05; ∗∗*p* ≤ 0.01; ∗∗∗*p* ≤ 0.001; ns, no significance; #, statistical reference. All statistical information may also be provided in form of a source data file.

## Supplemental information

Document S1. Figures S1–S8

Table S1. List of ligands

Table S2. Identified viral peptide fragments and IgG epitopes from patients with COVID-19 that match FPR activators

Table S3. Enrichment of aggregation hotspots in FPR1 and neutrophil activators
