# Supplementary material for: Fragments of viral surface proteins modulate innate immune responses via formyl peptide receptors
Source: iScience. 2025 Jun 30;28(8):113019. doi: 10.1016/j.isci.2025.113019 (PMC12284294; doi:10.1016/j.isci.2025.113019)
Supplement: Document S1. Figures S1–S8 [file mmc1.pdf]

iScience, Volume *28*

Supplemental information

Fragments of viral surface proteins modulate innate immune responses via formyl peptide receptors

Heiko Heilmann, Lukas Busch, Celine Buchmann, Islam Mohamed, Adrian Theiß, Sabryna Junker, Stefan Lohse, and Bernd Bufe

Supplemental Figures

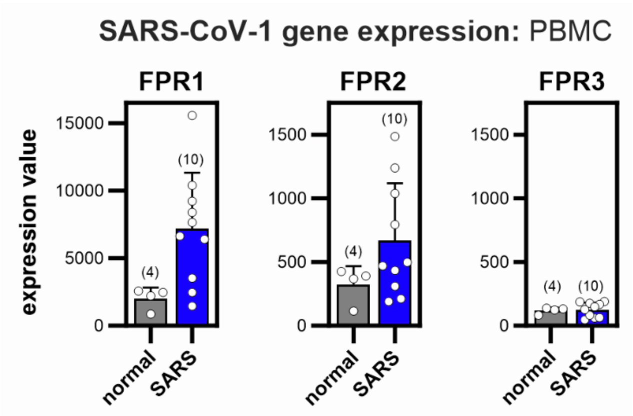

Figure S1: FPR1, FPR2 and FPR3 gene expression in PBMCs of SARS-CoV patients. PBMC gene expression data of SARS-CoV patients (blue; n=10) and healthy control individuals (grey; n=4). Expression values were gathered from NCBI (GEO Dataset: accession: GDS1028, ID: 1028; GEO Profile: FPR1 ID: 9264589, FPR2 ID: 9267790, FPR3 ID: 9268454). Data are represented as mean +/- SD.

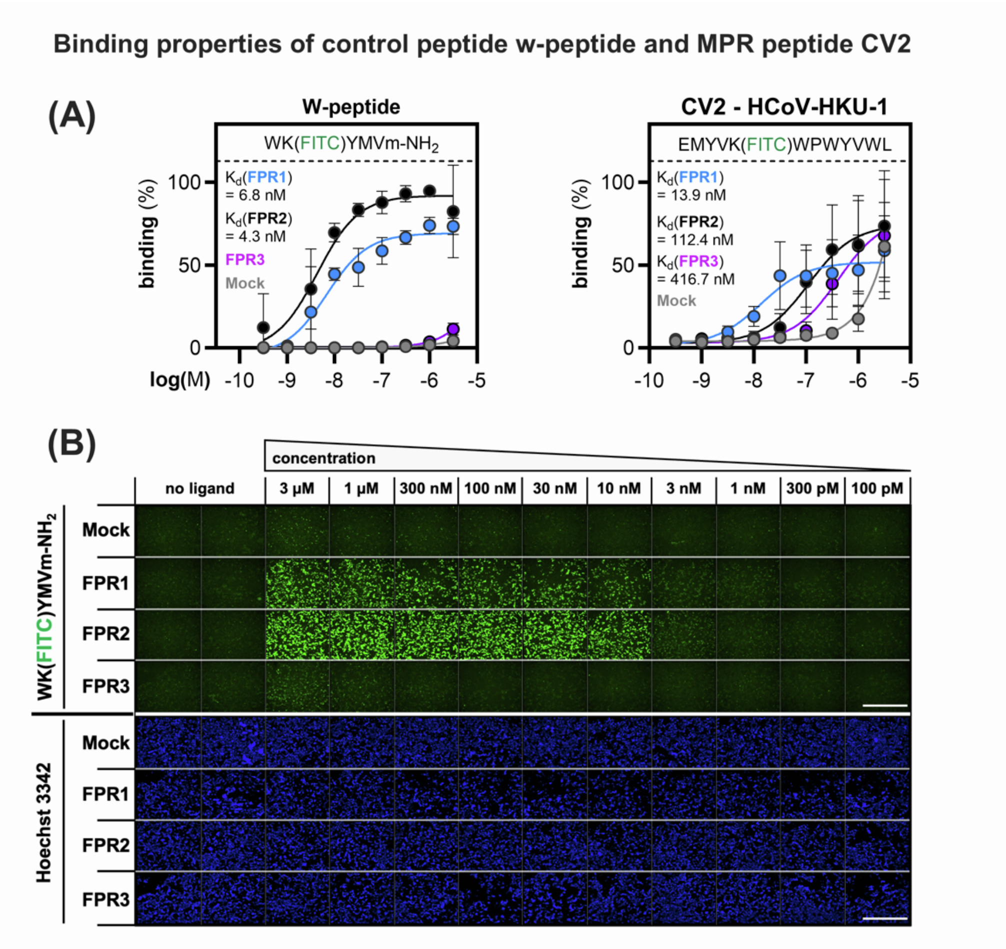

Figure S2: Binding properties of full-length MPR peptide CV2 and control peptide W-peptide. **(A)** Binding kinetics of FITC-labeled W-peptide (left) and CV2-FITC (right) on HEK293T cells transfected with either FPR1, FPR2, FPR3 or Mock plasmid. Graphs display mean values of three independent experiments (n=3) that were normalized to the largest value of the respective dataset. Data are represented as mean +/- SD. **(B)** High-throughput image of a representative experiment that displays W-peptide binding as quantified in (A). Scale bars represents 1000 µm.

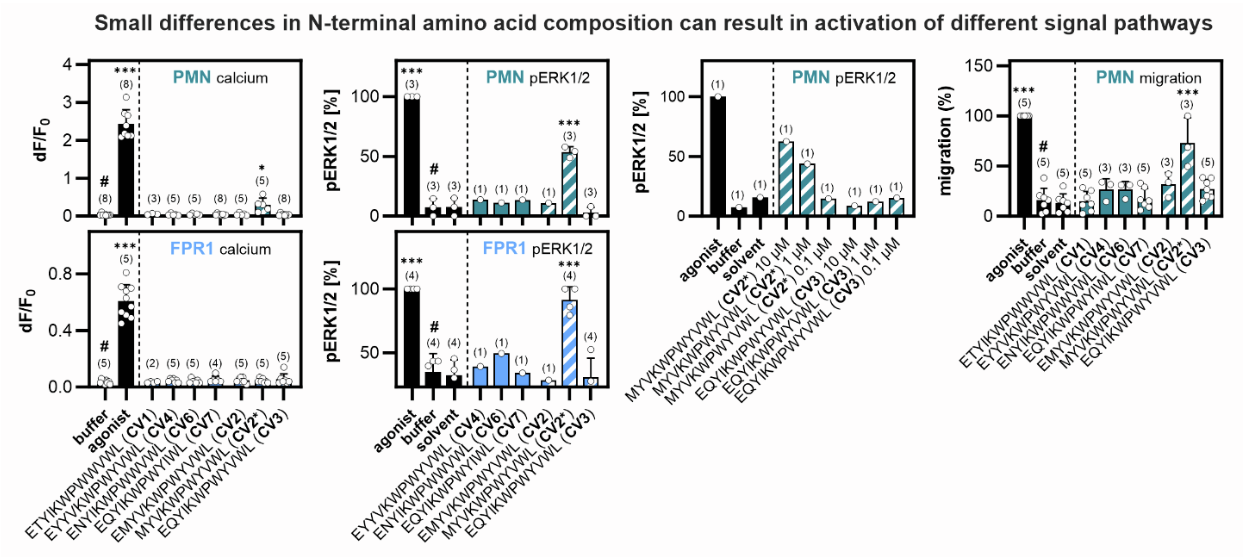

Figure S3: MPR peptide length and amino acid sequence can influence the signaling pathways in neutrophils in an FPR1-dependent manner.

Responses of neutrophils (top) and FPR1 transfected HEK293T cells (bottom) towards MPR peptide application (10 µM). Left: calcium signals (PMN, n=3-8; FPR1/HEK293T, n=2-5), Middle left: ERK1/2 phosphorylation (PMN, n=1-3; FPR1/HEK293T, n=1-4). Middle right: neutrophil concentrations dependent ERK1/2 phosphorylation (n=1). Data were normalized to the 10 µM W-peptide responses to compensate for differences in the maximal signals. Right: neutrophil migration (n=3-5). Data from different donors were normalized to the f-MLF responses (10 nM) to compensate individual differences in the total responses. Negative control: buffer; positive control W-peptide (10 µM). Data are represented as mean +/- SD. Statistics were performed using Dunnett's post-hoc one-way ANOVA to a reference value that is indicated by **#**. Significances are $*p \leq 0.05$; $**p \leq 0.01$; $***p \leq 0.001$; ns, no significance.

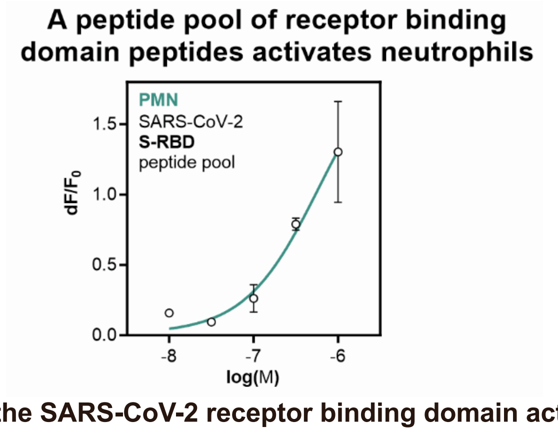

Figure S4: Peptides from the SARS-CoV-2 receptor binding domain activate neutrophils.

Dose-dependent calcium flux of neutrophils in response to a peptide pool that comprises peptides derived solely from the receptor binding domain of the SARS-CoV-2 spike protein (n=3). Data are represented as mean +/- SD.

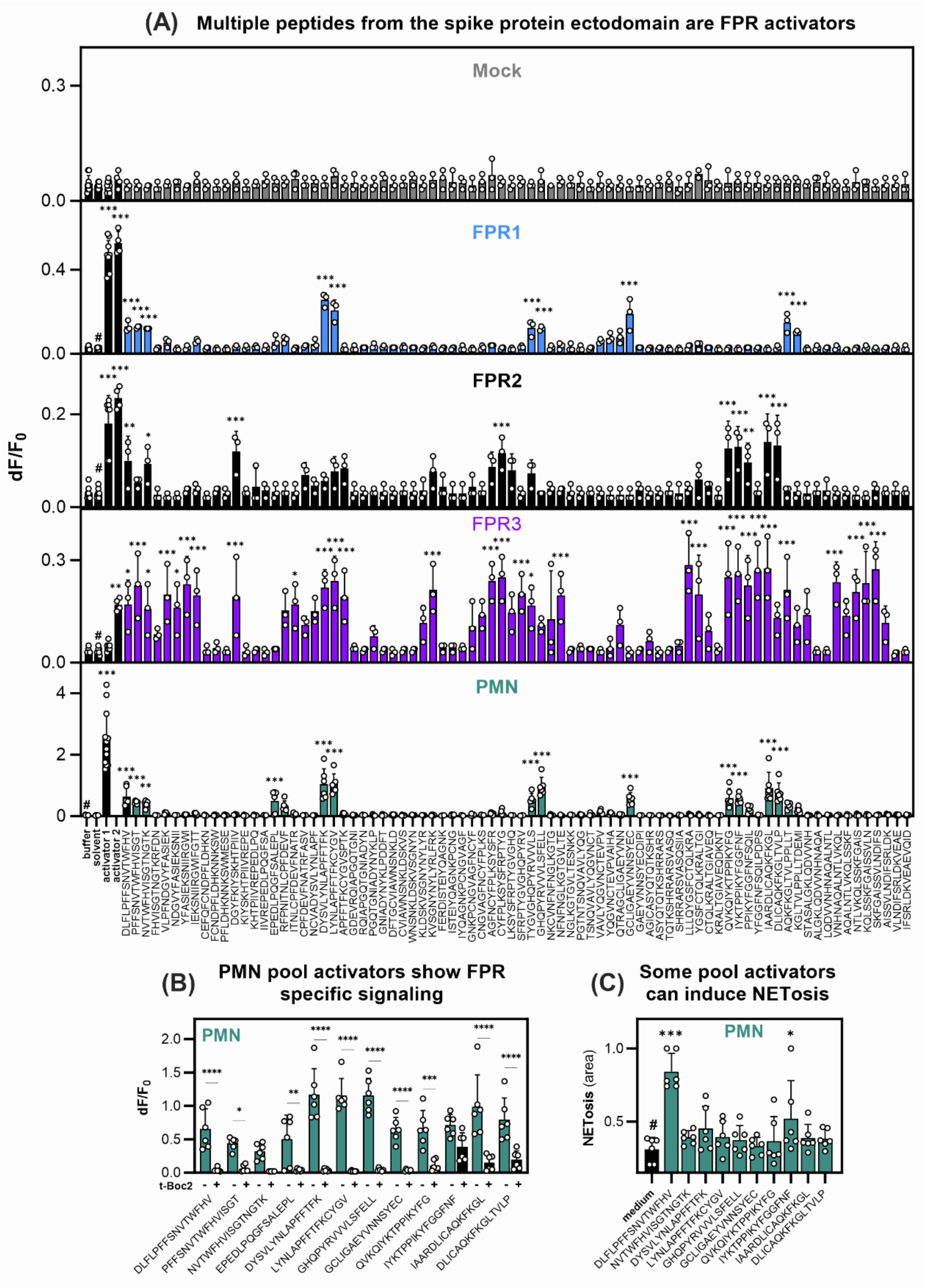

Figure S5: SARS-CoV-2 peptide pool screening and neutrophil response to spike protein activators.

(A) Bar graphs corresponding to the heatmap values in Fig. 4C showing the calcium responses of FPR1-, FPR2-, FPR3- or Mock-transfected HEK293T cells (n=3) as well as human polymorphonuclear granulocytes (PMN; n=6) in response towards a library of 80 SARS-CoV omicron variant B.1.1.529 spike protein pool derived peptides (10 µM). Negative controls represent the appropriate assay buffer (PMNs, ringer solution; HEK293T, C1 assay buffer) and solvent (DMSO in equal amounts to ligand dilution). Activator 1 (W-peptide, 10 µM) served as positive control for FPR1/FPR2 transfected HEK293T cells and neutrophils. Activator 2 denotes the FPR3 activator WKYMVm-CHO (10 µM; not tested on neutrophils). **(B)** Inhibition of neutrophil calcium flux mediated by selected pool activators (10 µM) with FPR1/2 inhibitor t-Boc2 (10 µM) (n=6). **(C)** Neutrophil NETosis mediated by the treatment with selected spike protein activators (30 µM) (n=3). NETosis after 4 h was measured by area and normalized to the largest value of the individual dataset to compensate for differences in the maximal signals. Data are represented as mean +/- SD. Statistics were performed using Dunnett's post-hoc one-way ANOVA to a reference value that is indicated by **#**. Significances are $*p \leq 0.05$; $**p \leq 0.01$; $***p \leq 0.001$; ns, no significance.

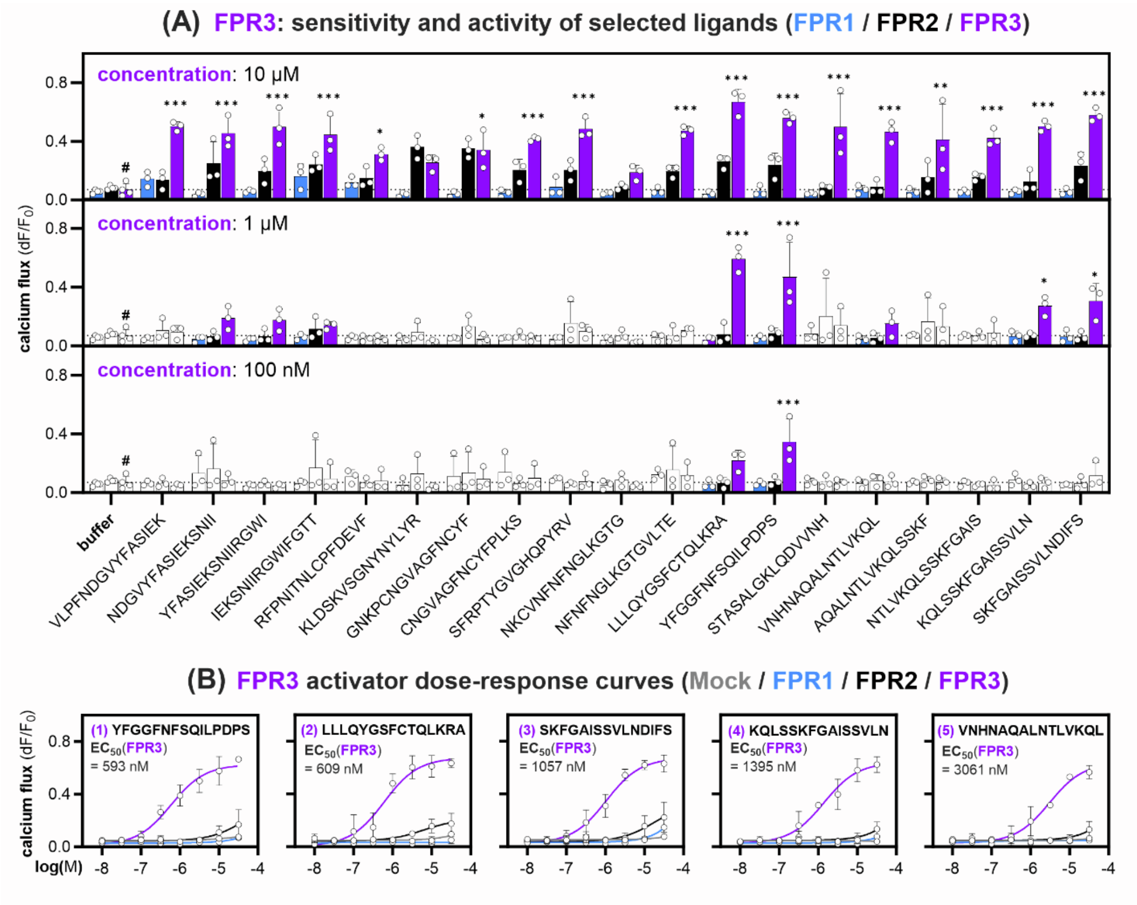

Figure S6: Sensitivity of FPR3-selective ligands from the SARS-CoV-2 spike protein.
(A) Calcium mobilization of FPR1-, FPR2-, FPR3- or Mock-transfected HEK293T cells in response towards selected ligands that were identified in the library screening of SARS-CoV-2 omicron variant B.1.1.529 spike protein pool derived peptides in Fig. 4C (n=3). The peptides were applied in the concentrations 10 µM (top), 1 µM (middle), 100 nM (bottom) and shown colorized if FPR3 signal response exceeds negative controls threshold and signaling intensities of FPR1 and FPR2. Negative controls represent C1 assay buffer. **(B)** Dose-dependent calcium flux of FPR1-, FPR2-, FPR3- or Mock-transfected HEK293T cells in response towards the most sensitive FPR3-selective ligands of the tests in (A) (n=3). Data are represented as mean +/- SD. Statistics were performed using Dunnett's post-hoc one-way ANOVA to a reference value that is indicated by **#**. Significances are $*p \leq 0.05$; $**p \leq 0.01$; $***p \leq 0.001$; ns, no significance.

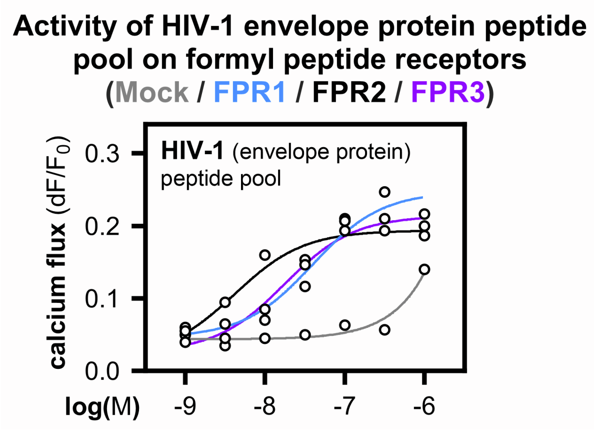

Figure S7: Activity of HIV-1 envelope protein peptide pool on formyl peptide receptors.
Dose-dependent calcium mobilization of FPR1-, FPR2-, FPR3- or Mock-transfected HEK293T cells in response to a peptide pool that comprises 150 peptides derived from the envelope protein of HIV-1 (n=3). Data are represented as mean signals.

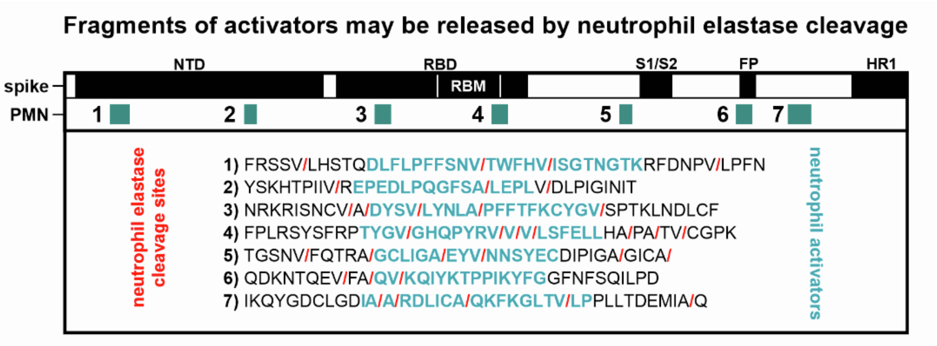

Figure S8: Spike protein cleavage by neutrophil elastase may release FPR activators.
Analysis of the seven activator regions in the SARS-CoV-2 Omicron Spike B.1.1.529 (PDB: 7QO7; data of peptide screening in Fig. 4). Neutrophil elastase cleavage sites in the activator regions are indicated as /. Cleavage sites were determined by SwissProt/Expasy tool PeptideCutter.
